# Supplementary material for: Long-term memory is formed immediately without the need for protein synthesis-dependent consolidation in Drosophila
Source: Nat Commun. 2019 Oct 7;10:4550. doi: 10.1038/s41467-019-12436-7 (PMC6779902; doi:10.1038/s41467-019-12436-7)
Supplement: Supplementary file 1 — Supplementary Information [file 41467_2019_12436_MOESM1_ESM.pdf]

Supplementary Materials for

Long-term memory is formed immediately without the need for protein synthesis-dependent consolidation in *Drosophila*

Bohan Zhao, Jiameng Sun, Xuchen Zhang, Han Mo, Yijun Niu, Qian Li, Lianzhang Wang, Yi Zhong*

*Corresponding author: zhongyi@tsinghua.edu.cn.

This PDF file includes:

Supplementary Figures 1 to 6
Supplementary Table 1

Supplementary Figure 1

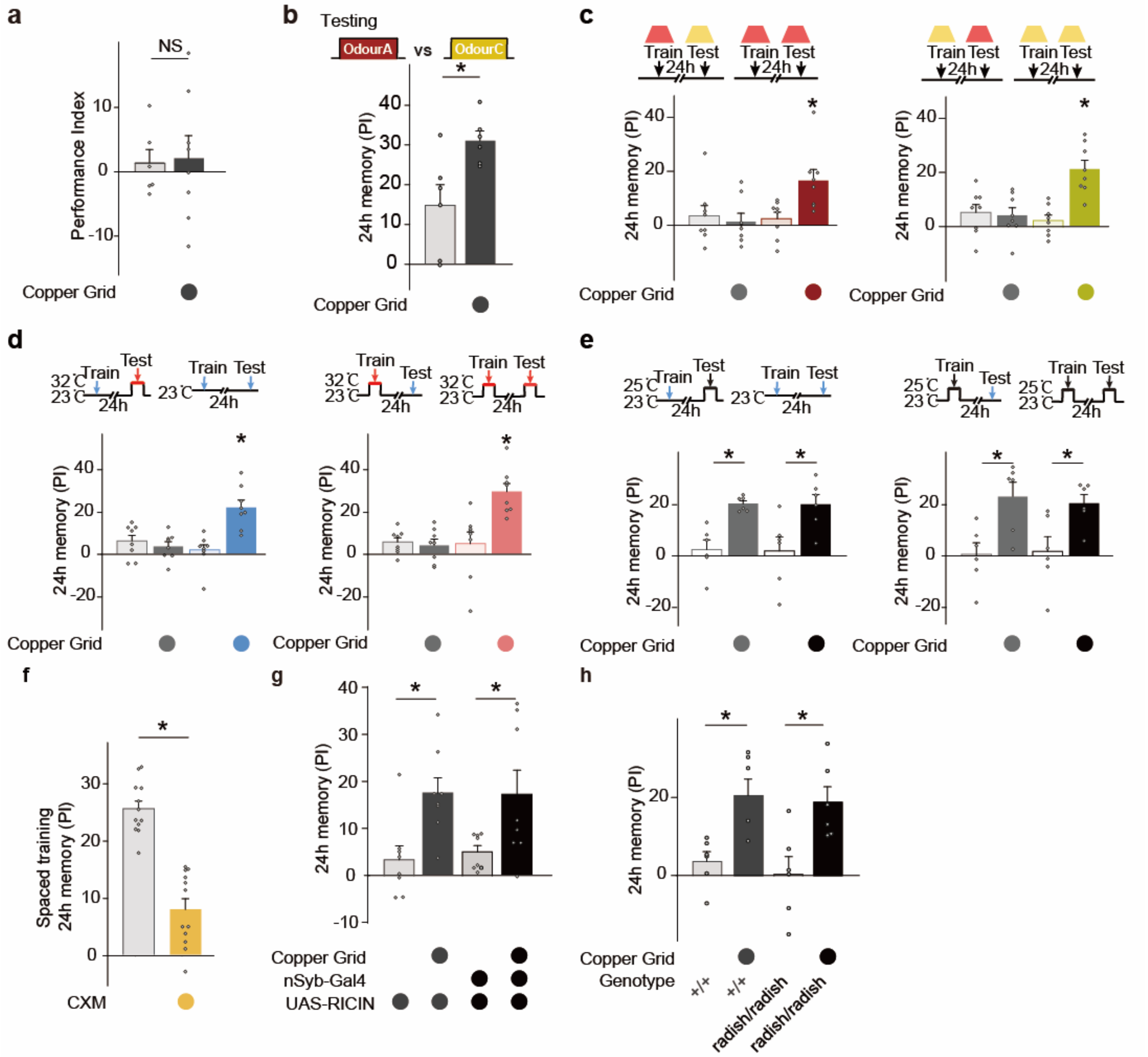

Supplementary Figure 1. Further identification of cLTM (Related to Figure 1)

(a) Naïve flies perform no memory with copper grid (n = 6–8). (b) Left: protocol. Right: Copper grid improves 24 h memory performance between conditioned odour and new odour. (c) Changing the light color from red to yellow abolishes context-dependent long-term memory (cLTM), and vice versa (n = 8). (d) Changing the temperature from 23℃ to 32℃ abolishes cLTM, and vice versa (n = 8). (e) Changing the temperature from 23℃ to 25℃ does not impair cLTM, and vice versa (n = 8). (f) Long-term memory formed

after multiple-spaced training is impaired after feeding with cycloheximide (n = 12). (g) Inhibiting protein synthesis with RICIN immediately after training cannot destroy cLTM (n = 12). (h) cLTM was not impaired in *radish* mutant (n = 6). Data are mean performance indices ± SEM; individual data points are displayed as dots; *P < 0.05 by ANOVA or t-test.

Supplementary Figure 2

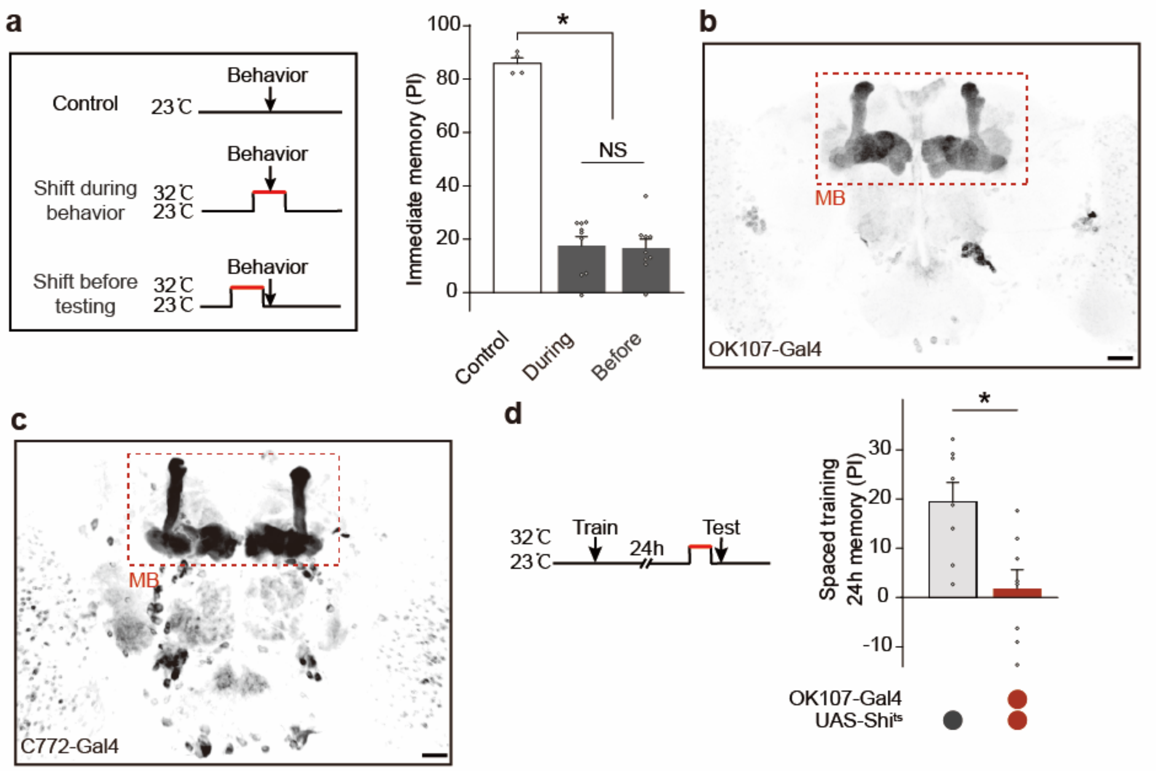

Supplementary Figure 2. Further investigate the role of MB (Related to Figure 2)

(a) Left: protocols. Right: performance of immediate memory of OK107-Gal4;UAS-Shi^{ts} under different conditions (n = 6). The blocked behaviour performances of crossed fly were the same during or after heat shock. (B and C) OK107-Gal4 and C772-Gal4. The mushroom body is broadly labeled. Scale bar = 20 μm. (d) Blockade of OK107 neurons impairs long-term memory after multiple-spaced training (n = 8). Data are mean performance indices ± SEM; individual data points are displayed as dots; *P < 0.05 by ANOVA or t-test.

Supplementary Figure 3

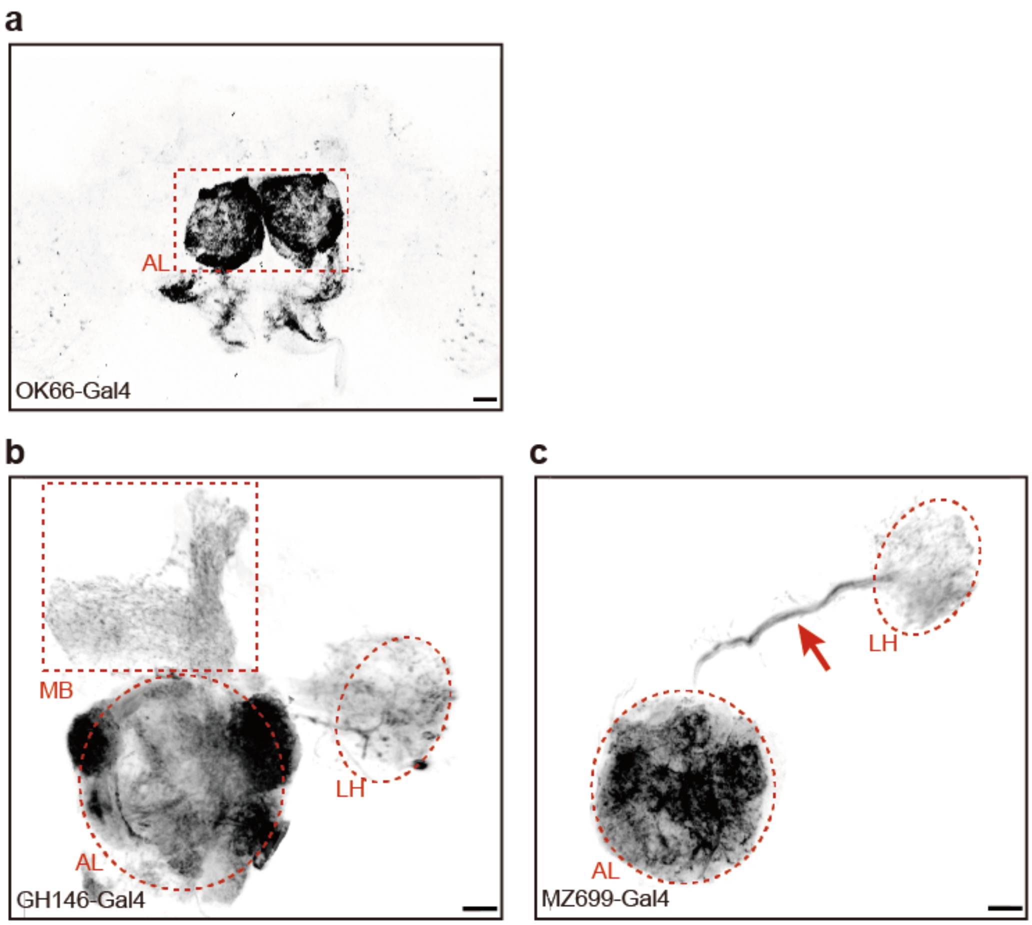

Supplementary Figure 3. Expression patterns of Gal4 lines used in Figure 3 (Related to Figure 3)

(a) OK66-Gal4. The antennal lobe is broadly labeled. (b) GH146-Gal4. The excitatory projection neurons are broadly labeled. (c) MZ699-Gal4. The inhibitory projection neurons are broadly labeled. Scale bar = 20 μm.

Supplementary Figure 4

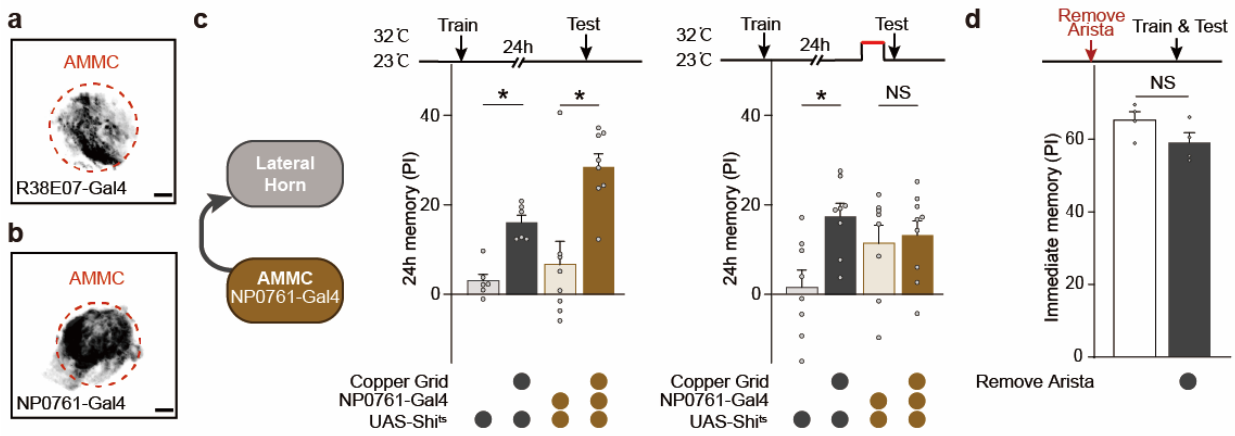

Supplementary Figure 4. Further investige the role of AMMC (Related to Figure 4)

(a and b) R38E07-Gal4 and NP0761-Gal4. The AMMC is broadly labeled. Scale bar = 20 μm. (c) Blockade of NP0761 neurons abolishes cLTM retrieval (n = 8). (d) Removal of the arista cannot impair immediate memory (n = 3). Data are mean performance indices ± SEM; individual data points are displayed as dots; *P < 0.05 by ANOVA or t-test.

Supplementary Figure 5

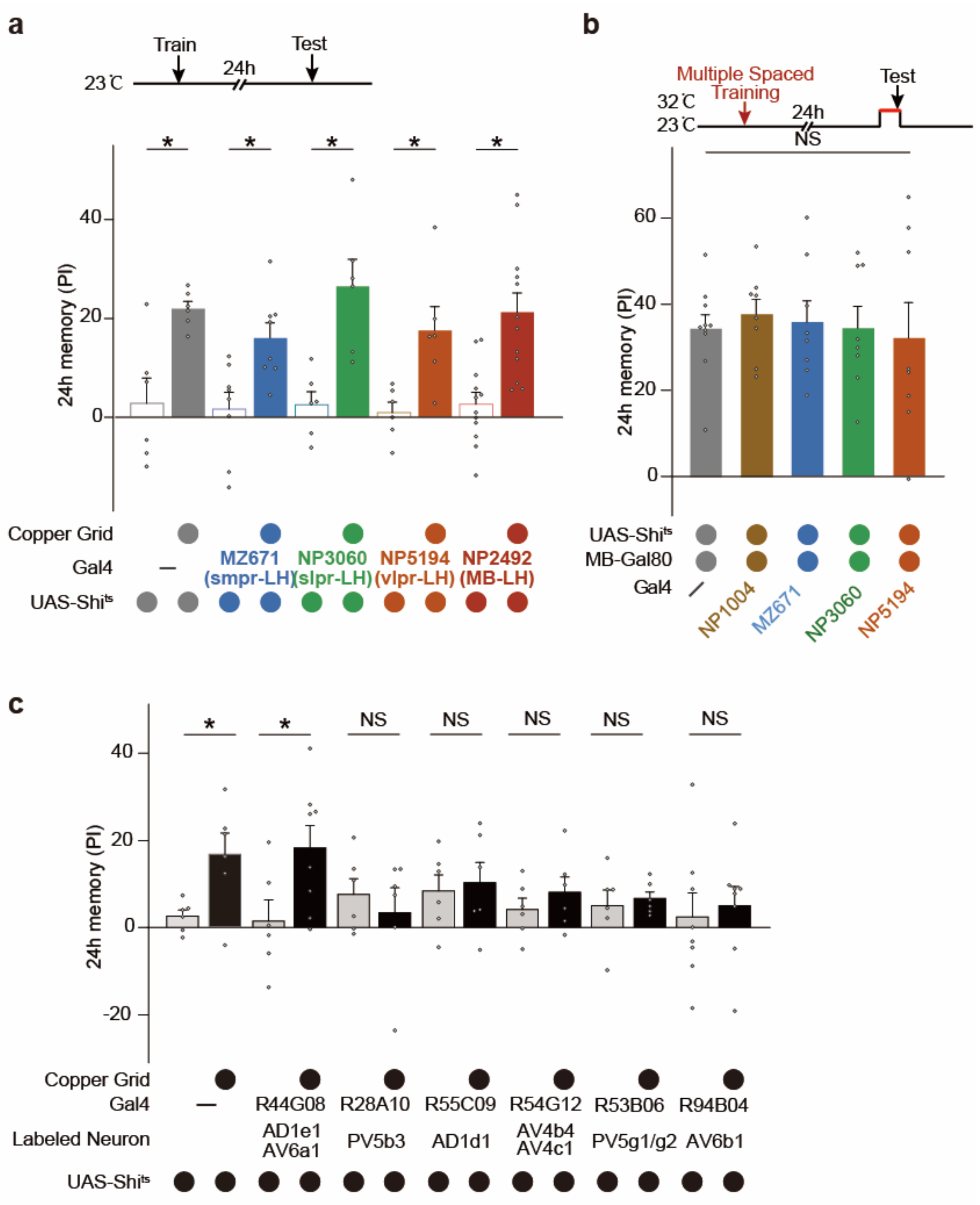

Supplementary Figure 5. Further investige the role of LH (Related to Figure 5)

(a) Top: protocol. Bottom: Crosses performs normally without blockade of LH neurons. (n = 8). (b) Top: protocol. Bottom: Crosses performs normal LTM with blockade of LH neurons during retrieval. (n = 8). (c) Blocking LH output neurons with R28A10, R55C09,

R54G12, R53B06, and R94B04-Gal4s during retrieval impaires cLTM, while R44G08-Gal4 does not (n = 6). Data are mean performance indices ± SEM; individual data points are displayed as dots; *$P < 0.05$ by ANOVA or t-test.

Supplementary Figure 6

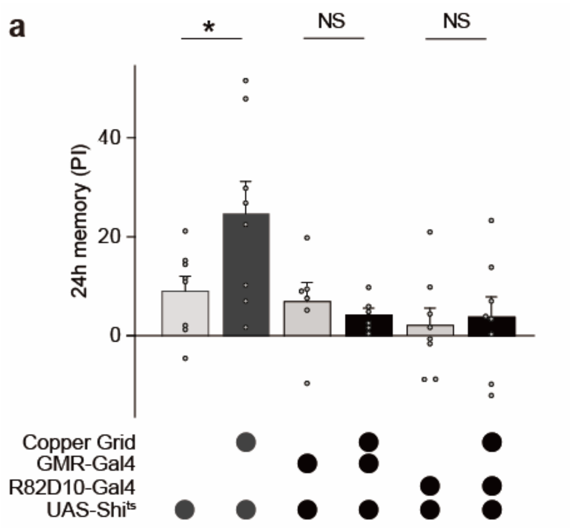

Supplementary Figure 6. Further investige the role of visual system (Related to Figure 6)

(a) Blocking eyes with GMR-Gal4 and the optic lobe with R82D10-Gal4 during retrieval both impaired cLTM. (n = 6–8). Data are mean performance indices ± SEM; individual data points are displayed as dots; $*P < 0.05$ by ANOVA or t-test.

Supplementary Table 1

Genotype	Context	Olfactory Acuity	
		**OCT (1.5×10^{-3})**	**MCH ($1.0\text{x}10^{-3}$)**
+/+	No Grid	37.673±3.483	37.539±4.222
	Copper Grid	35.475±5.360	44.478±2.889

Supplementary Table 1. Task-Relevant Sensorimotor Responses, Related to Figure 1

Sensorimotor responses to odours used in the conditioning task were tested. For all groups, a one-way ANOVA was performed. For odour sensitivity, comparisons between the No Grid and Copper Grid groups did not show any significant differences. Data are shown as mean ± SEM, n = 8.
