# Supplementary material for: Long-term memory is formed immediately without the need for protein synthesis-dependent consolidation in Drosophila
Source: Nat Commun. 2019 Oct 7;10:4550. doi: 10.1038/s41467-019-12436-7 (PMC6779902; doi:10.1038/s41467-019-12436-7)
Supplement: Supplementary file 3 — Reporting Summary [file 41467_2019_12436_MOESM3_ESM.pdf]

nature research

Corresponding author(s): Yi Zhong
Last updated by author(s): Jun 4, 2019

Reporting Summary

Nature Research wishes to improve the reproducibility of the work that we publish. This form provides structure for consistency and transparency in reporting. For further information on Nature Research policies, see Authors & Referees and the Editorial Policy Checklist .

Statistics

For all statistical analyses, confirm that the following items are present in the figure legend, table legend, main text, or Methods section.

n/a	Confirmed	
☐	☒	The exact sample size (n) for each experimental group/condition, given as a discrete number and unit of measurement
☐	☒	A statement on whether measurements were taken from distinct samples or whether the same sample was measured repeatedly
☐	☒	The statistical test(s) used AND whether they are one- or two-sided<br>*Only common tests should be described solely by name; describe more complex techniques in the Methods section.*
☐	☒	A description of all covariates tested
☐	☒	A description of any assumptions or corrections, such as tests of normality and adjustment for multiple comparisons
☐	☒	A full description of the statistical parameters including central tendency (e.g. means) or other basic estimates (e.g. regression coefficient) AND variation (e.g. standard deviation) or associated estimates of uncertainty (e.g. confidence intervals)
☐	☒	For null hypothesis testing, the test statistic (e.g. F, t, r) with confidence intervals, effect sizes, degrees of freedom and P value noted<br>*Give P values as exact values whenever suitable.*
☒	☐	For Bayesian analysis, information on the choice of priors and Markov chain Monte Carlo settings
☒	☐	For hierarchical and complex designs, identification of the appropriate level for tests and full reporting of outcomes
☒	☐	Estimates of effect sizes (e.g. Cohen's d, Pearson's r), indicating how they were calculated

Our web collection on statistics for biologists contains articles on many of the points above.

Software and code

Policy information about availability of computer code

Data collection	Zen2010B.
Data analysis	Zen2010B, Matlab 2017a, GraphPad Prism 5

For manuscripts utilizing custom algorithms or software that are central to the research but not yet described in published literature, software must be made available to editors/reviewers. We strongly encourage code deposition in a community repository (e.g. GitHub). See the Nature Research guidelines for submitting code & software for further information.

Data

Policy information about availability of data

All manuscripts must include a data availability statement. This statement should provide the following information, where applicable:
- Accession codes, unique identifiers, or web links for publicly available datasets
- A list of figures that have associated raw data
- A description of any restrictions on data availability

Provide your data availability statement here.

Field-specific reporting

Please select the one below that is the best fit for your research. If you are not sure, read the appropriate sections before making your selection.

☒ Life sciences ☐ Behavioural & social sciences ☐ Ecological, evolutionary & environmental sciences

For a reference copy of the document with all sections, see nature.com/documents/nr-reporting-summary-flat.pdf

Life sciences study design

All studies must disclose on these points even when the disclosure is negative.

Sample size	The sample size chosen throughout this study is same as others in the field.
Data exclusions	No data was excluded.
Replication	All attempts at replication were successful
Randomization	Different genotypes were compared in this study and each genotype is a group.
Blinding	Investigators were blinded during data analysis.

Reporting for specific materials, systems and methods

We require information from authors about some types of materials, experimental systems and methods used in many studies. Here, indicate whether each material, system or method listed is relevant to your study. If you are not sure if a list item applies to your research, read the appropriate section before selecting a response.

Materials & experimental systems

n/a	Involved in the study	
☐	☒	Antibodies
☒	☐	Eukaryotic cell lines
☒	☐	Palaeontology
☐	☒	Animals and other organisms
☒	☐	Human research participants
☒	☐	Clinical data

Methods

n/a	Involved in the study	
☒	☐	ChIP-seq
☒	☐	Flow cytometry
☒	☐	MRI-based neuroimaging

Antibodies

Antibodies used	anti-GFP (chicken, 1:2000; Abcam, Cambridge, UK), anti-nc82 (mouse, 1:10; Developmental Studies Hybridoma Bank, Iowa City, IA, USA), anti-DsRed (rabbit, 1:500; Takara Bio, Kyoto, JP), anti-chicken (Alexa Fluor 488, 1:200), anti-mouse (Alexa Fluor 647, 1:200) and anti-rabbit (Alexa Fluor 647, 1:200).
Validation	anti-GFP: Immunofluorescence, Species: Drosophila<br>anti-NC82: Immunofluorescence, Species: Drosophila<br>anti-DsRed: Immunofluorescence, Species: Drosophila

Animals and other organisms

Policy information about studies involving animals; ARRIVE guidelines recommended for reporting animal research

Laboratory animals	Drosophila melanogaster: w1118(isoCJ1), UAS-Shibirets1, OK66-Gal4, GMR-Gal4, rut1, rut2080, radish, dumb2, MB-Gal80, NP1004-Gal4 (stock #112440), NP6099-Gal4 (#105125), NP3060-Gal4 (#104359), NP5194-Gal4 (#113625), NP2492-Gal4 (#104219), R38E07-Gal4 (stock #50007), nSyb-Gal4 (#51635), R82D10-Gal4 (#40145), R44G08-Gal4 (#50216), R28A10-Gal4 (#48074), R55C09-Gal4 (#39107), R54G12-Gal4 (#41280), R53B06-Gal4 (#38863), R94B04-Gal4 (#40675), UAS-GCamP6f (#42747), UAS-syt::GFP (#6925), UAS-p65aD::CaM;UAS-MKII::nlsLexADBDo;LexAop2-mCD8::GFP;UAS-mCD8::RFP (#62827), UAS-dDA1-RNAi (TH04278.N), GH146-Gal4, MZ671-Gal4, UAS-RICIN, NP0761-Gal4, MZ699-Gal4 and UAS-dCREB2b.
Wild animals	The study did not involve wild animals
Field-collected samples	The study did not involve samples collected from the field.
Ethics oversight	Ethics oversight is not required for Drosophila investigations.

Note that full information on the approval of the study protocol must also be provided in the manuscript.
